# Could human coronavirus OC43 have co-evolved with early humans?

**DOI:** 10.1590/1678-4685-GMB-2017-0192

**Published:** 2018-06-28

**Authors:** Paulo Eduardo Brandão

**Affiliations:** 1 Departmento de Medicina Veterinaria Preventiva e Saúde Animal, Faculdade de Medicina Veterinária e Zootecnia, Universidade de São Paulo, São Paulo, SP, Brazil

**Keywords:** Codon usage, coronavirus, spillover, coevolution

## Abstract

This paper reports on an investigation of the role of codon usage evolution on the suggested bovine-to-human spillover of Bovine coronavirus (BCoV), an enteric/respiratory virus of cattle, resulting in the emergence of the exclusively respiratory Human coronavirus OC43 (HCoV-OC43). Analyses based on full genomes of BCoV and HCoV-OC43 and on both human and bovine mRNAs sequences of cholecystokinin (CCK) and surfactant protein 1 A (SFTP1-A), representing the enteric and respiratory tract codon usage, respectively, have shown natural selection leading to optimization or deoptimization of viral codon usage to the human enteric and respiratory tracts depending on the virus genes under consideration. A higher correlation was found for the nucleotide distance at the 3^rd^ nucleotide position of codons and codon usage optimization to the human respiratory tract when BCoV and HCoV-OC43 were compared. An MCC tree based on relative synonymous codon usage (RSCU) data integrating data from both viruses and hosts into a same analysis indicated three putative host/virus contact dates ranging from 1.54E8 to 2.44E5 years ago, suggesting that an ancestor coronavirus might have followed human evolution.

## Introduction

Human coronavirus OC43 (*Nidovirales: Coronaviridae: Coronavirinae: Betacoronavirus: Betacoronavirus 1*, HCoV-OC43) is an epitheliotropic respiratory virus widespread in human populations and involved in common cold (Mäkelä *et al.*, 1998), while Bovine coronavirus (BCoV), another host-type of *Betacoronavirus 1*, is commonly found infecting both the respiratory and enteric tracts of cattle and might lead to respiratory disease and diarrhea/dysentery (Dea *et al.*, 1995; Saif, 2010). A suggested bovine-to-human spillover of BCoV resulting in HCoV-OC43 has been proposed around year 1890, based on the spike (S) gene sequences of BCoV and HCoV-OC43 (Vijgen *et al.*, 2005b; Bidokhti *et al.*, 2013).

The *Betacoronavirus 1* genome is a ca. 32 kb single-stranded positive-sense 5’ capped RNA coding for subgenomic mRNAs (sgmRNAs) in the order ORF1(replicase)-HE (hemagglutinin-esterase)-S-(spike glycoprotein)-E (envelope protein)-M (membrane protein)-I (internal protein)-N (nucleocapsid protein). A 32kDa accessory protein (ns2) is found in both BCoV and HCoV-OC43 where the gene (ns2) is located before the HE gene (Masters, 2006; Labonté *et al.*, 1995). The replicase polyprotein is cleaved into 16 non-structural proteins (nsps) with multiple roles in sgmRNA synthesis and genome replication (Ziebuhr and Snijder, 2007).

Betacoronaviruses have a history of spillover to humans leading to the emergence of pathogens, such as the Middle East Respiratory Syndrome Human Coronavirus (MERS-CoV) and the Severe Acute Respiratory Syndrome Human Coronavirus (HCoV-SARS) (Li *et al.*, 2005; Gossner *et al.*, 2016). Such a pathogen emergence is limited by ecological and genetic factors (Gandon *et al.*, 2013), and codon usage, *i.e.*, the deviation from the random use of different codons for the 2 to 6-fold degenerate codons (Hershberg and Petrov, 2009; Roth *et al.*, 2012), is one genetic factor that might help to explain this process.

Codon usage evolution has a measurable role on the adaptation of viruses to hosts (Chantawannakul and Cutler, 2008) due to natural selection based on translation efficiency and also drift according to the genomic mutation pressure (Nei and Kumar, 2000; Hershberg and Petrov, 2009). Nonetheless, codon usage studies meet limitations on plausible indicators and dating methods to estimate the coevolution patterns after a virus meets a new host species. If the dating of a spillover event based solely on virus nucleotide sequence data would agree with codon usage dating, based on both virus and host data, is hitherto unknown.

The aim of this study was to analyze the BCoV/HCoV-OC43 spillover to humans based on codon usage data for codon selection regime, fitness and virus/host relationship dating estimates.

## Materials and Methods

### Sequences

Complete genome sequences were retrieved from GenBank for BCoV (strain BCoV R-AH187, EF424620.1), detected in 2000 in the USA (Zhang *et al.*, 2007), and HCoV-OC43 (strain 19572, AY903460.1), detected in 2004 in Belgium (Vijgen *et al.*, 2005a). These two sequences were considered as representatives of the diversity of each virus, and the inclusion criteria were based on genome completeness and annotation.

Further human coronaviruses complete genome sequences included HCoV-HKU1 (KF686341.1), HCoV-NL63 (DQ445911.1), HCoV-229E (JX503061.1), HCoV-SARS (AY291315), and two HCoV-MERS (KJ156949 from a strain detected in a human patient and KJ713299.1 detected in a dromedary camel).

The eight coronavirus genomes were split into each coding region/mRNA for the analyses. Nsps 1-16 sequences were checked based on nsps 3 and 5 cleavage sites (Ziebuhr and Snijder, 2007; Wojdyla *et al.*, 2010).

As representatives of highly expressed, tissue-specific proteins for the respiratory and enteric tracts of *H. sapiens sapiens* and *B. taurus taurus*, complete mRNA sequences were retrieved from GenBank for the surfactant protein A1 SFTPA1 (NM_001077838.2 and NG_021189.1) and cholecystokinin CCK (NM_001046603.2 and NM_000729.4), respectively.

### Codon adaptation index (CAI) limits for human coronaviruses and ΔCAI for HCoV-OC43 and BCoV

CAI is an indicator of translational fitness of an mRNA regarding a reference translational system, ranging from 0 (no fitness) to 1 (highest fitness) (Lee *et al.*, 2010). To determine the lower and upper limits for HCoVs in the respiratory and enteric tracts of humans, the eight HCoV sequences had their CAIs calculated for each coding region/mRNA using human SFTPA1 and CCK sequences as references in CAI Calculator 2 (Wu *et al.*, 2005) based on the equation by Sharp and Li (1987).

CAI differences (ΔCAI) were calculated as HCoV-OC43 CAI - BCoV CAI (calculated as mentioned above) for each coding region/mRNA regarding human respiratory and enteric tracts in order to access the codon optimization (ΔCAI>1) or deoptimization (ΔCAI<1) for the bovine-to-human spill over.

### Codon usage selection regimes

For each HCoV-OC43 and BCoV coding region/mRNA, the observed effective number of codons (*Nc*) and the frequency of G or C at the 3rd codon positions in synonymous codons (%GC3s) (Wright, 1990) was calculated using ACUA 1.0 software (Vetrivel *et al.*, 2007) and CAI Cal (Puigbo *et al.*, 2008), and both indicators were plotted in the expected number of codons (ENC)/ expected %GC3 graph (Wright, 1990). Dots from observed values outside the expected values curve are an indication of natural selection, while those on the curve indicate drift/ mutation pressure.

### Viruses/hosts codon usage co-evolution analysis

For each HCoV-OC43 and BCoV coding region/mRNA and human and bovine CCK and SFTPA1, the values of RSCU (relative synonymous codon usage) were estimated for the 59 nonstop degenerate codons using Mega 7 software (Kumar *et al.*, 2016). Codons with RSCU <1 are considered non-preferred, and those with RSCU >1 are preferred, while an RSCU=1 indicates a neutral codon (Su *et al.*, 2009).

Next, continuous RSCU values were assigned the binary values 0 (RSCU≤1) and 1 (RSCU>1), and data from both hosts and both HCoV-OC43 and BCoV assembled into a single alignment were used to build an MCMC MCC tree with the simple model. This included estimated frequencies, burn in=10% states, uncorrelated exponential relaxed clock (which showed a lower standard deviation when compared to lognormal clock) and constant population size (due to the lack of consensus priors for an exponential growth coalescent analysis for *H. sapiens sapiens*, *B. taurus taurus* and coronaviruses) and was built using Beast v. 1.8.3 (Drummond and Rambaut, 2007; Drummond *et al.*, 2012).

Calibration times to estimate branch lengths were based on dates with 2004 (HCoV-OC43 strain 19572 detection date) as the reference year and were as follows: 200,000 years ago (y.a.) for *H. sapiens sapiens* (Weaver, 2012), 10,000 y.a. for *B. taurus taurus* based on the domestication dates for this species (reviewed by Ajmone-Marsan *et al.*, 2010), 114 y.a. for HCoV-OC43 (Vijgen *et al.*, 2005b) and 602 y.a. for BCoV based on the *Betacoronavirus 1* split (Lau *et al.*, 2015).

The RSCU binary distance between human and bovine CCK and SFTPA1 was calculated as the total difference for each of these two datasets and used as a measure of codon usage distance for the enteric and respiratory tracts, respectively, for these two host species.

## Results

### Codon adaptation index (CAI) limits for human coronaviruses and ΔCAI for HCoV-OC43 and BCoV

CAI upper and lower limits for the seven human coronaviruses included in this study in human respiratory and enteric tracts were 0.244-0.611 (corresponding to HCoV-SARS nsp11 and nsp10, respectively) and 0.244-0.472 (corresponding to HCoV-SARS ORF7b and nsp11, respectively).

CAI optimization (ΔCAI>1) was found for nsp2-5, nsp8, nsp11, nsp15, ns2, HE, S, M, I and N and nsp2, nsp4-6, nsp11, nsp14, nsp16, ns2, M and N proteins on the enteric and respiratory tracts, respectively. Deoptimization (ΔCAI<1) was found nsp1, nsp6, nsp9-10, nsp12-14, nsp16 and E and nsp1, nsp3, nsp8-10, nsp12-13, HE, S, E and I proteins for the enteric and respiratory tracts, respectively.

**Figure 1:**
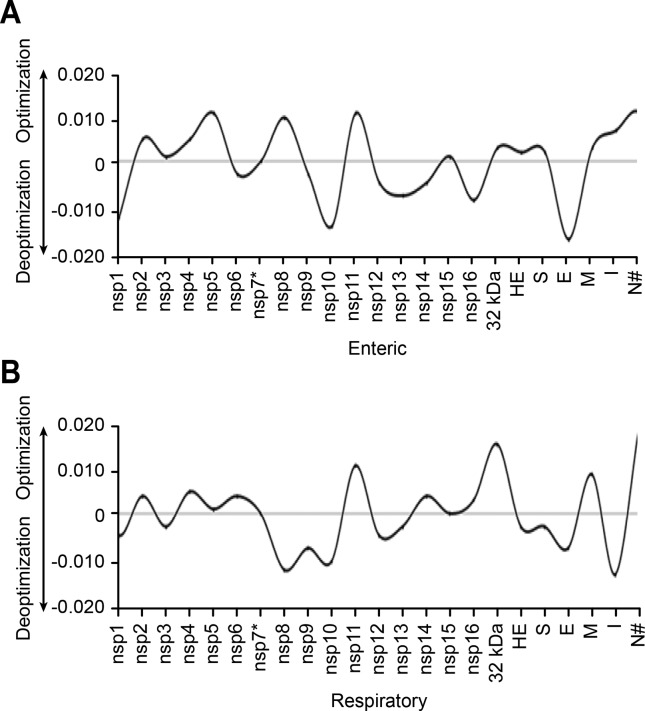
ΔCAI for BCoV and HCoV-OC43 coding regions/mRNAs for nsps1-16, ns2 and structural proteins HE, S, E, M, I and N regarding (A) human cholecystokinin (CCK) and (B) human surfactant protein A1 (SFTPA1) mRNAs as highly expressed, tissue specific proteins from the enteric and respiratory tracts, respectively. Positive ΔCAI values indicate viral codon usage optimization, while negative values indicate deoptimization. *=lowest distance from HCoVs lower CAI limit for both HCoV-OC43 and BCoV; #=highest distance from HCoVs lower CAI limit for both HCoV-OC43 and BCoV.

A ΔCAI=0 was found for nsp7 on both respiratory and enteric human tracts and for nsp15 on the respiratory tract. ΔCAI values for each coding region/mRNA of HCoV-OC43 on the human enteric and respiratory tracts are represented in Figure 1.

For both BCoV and HCoV-OC43 nsp7, the lowest CAI distance (-0.039) was found for both the human and respiratory and enteric tracts regarding the lower CAI limit calculated for all seven human coronaviruses, while the highest CAI distances for the lower human coronaviruses CAI was found for BCoV and HCoV-OC43 N for both the human and respiratory and enteric tracts (-0.282 and -0.302, respectively) and BCoV nsp15 (-0,282) for the human respiratory tract.

Correlation analysis of ΔCAI and nucleotide identities amongst the 23 BCoV and HCoV-OC43 homologous coding regions/ mRNAs based on 1st, 2nd and 3rd and on the 3rd nucleotide position only showed the highest r^2^ (correlation coefficient) value (0.27) for the 3rd nucleotide position regarding the human respiratory tract, while r^2^ values for ΔCAI and 1st, 2nd and 3rd regarding the human enteric and respiratory tracts were both 0.05 and, regarding the 3rd positions only and the human enteric tract, 0.07.

### Codon usage selection regimes

**Figure 2:**
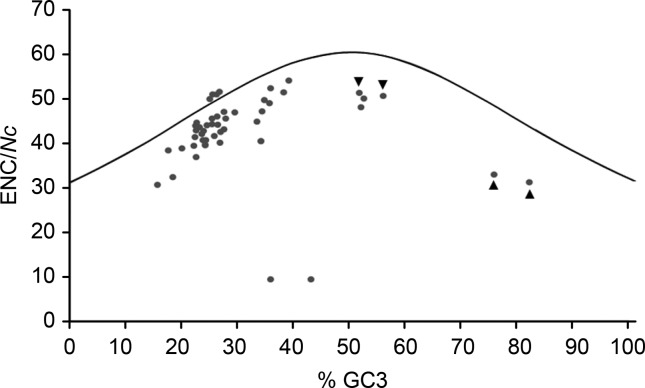
Observed (dots) and expected (curve) effective number of codons (*Nc* and ENC, respectively) on the Y axis and %GC3 on the X axis for BCoV and HCoV-OC43 coding regions/mRNAs for nsps1-16, ns2 and structural proteins HE, S, E, M, I and N and human and bovine CCK (left and right lower arrowheads, respectively) and SFTPA1 (right and left upper arrowheads, respectively) mRNAs.

All *Nc* x %GC3s plots were found either above or below the ENC x %GC3 expected curve for all HCoV-OC43 and BCoV coding regions/mRNAs and for human and bovine CCK and SFTPA1 (Figure 2), an indication that codon usage in these cases was ruled by natural selection.

In Figure 2, the two closest dots to bovine and human SFTPA1 dots represent the internal I protein of BCoV (upper) and HCoV-OC43 (lower), while the two dots at the bottom of the graph refer to BCoV and HCoV-OC43 nsp11.

### Viruses/hosts codon usage co-evolution analysis

**Figure 3:**
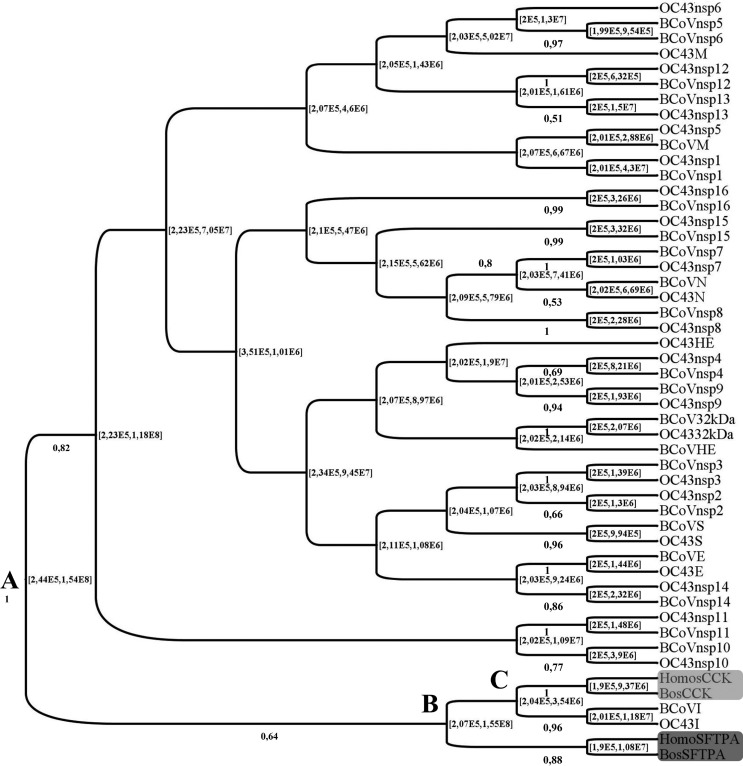
MCC tree (burn in = 10% states, uncorrelated exponential clock and constant population size) for BCoV and HCoV-OC43 coding regions/mRNAs for nsps1-16, ns2 and structural proteins HE, S, E, M, I and N and the representatives (mRNAs) of human and bovine highly-expressed, tissue specific proteins CCK (light grey rectangle, cholecystokinin, enteric tract) and SFTPA1 (dark grey rectangle, surfactant protein A1, respiratory tract) based on the respective binary RSCU (Relative synonymous codon usage) values, showing the host/viruses split nodes A, B and C. Values to the left of each node are the posterior probabilities (only values > 50 are shown) and values to the right of each node are the 95% HPD (in years since 2004, the date of the HCoV-OC43 strain 19572 detection).

All 95% HPDs (Highest Posterior Densities) are presented in years. In the MCC tree shown in Figure 3, the first split event (node A, 95% HPD 2.44E5-1.54E8) resulted in two major clusters, the largest one containing all HCoV-OC43 and BCoV coding regions/ mRNAs data except for I protein and a minor cluster containing both human and bovine CCK and SFTPA1 and HCoV-OC43 and BCoV I protein. For this minor cluster containing both hosts and coronaviruses codon usage statuses, a second split was found (node B, 95% HPD 2.07E5-1.55E8), resulting in a cluster with SFTPA1 only and another cluster with CCK and HCoV-OC43 and BCoV I, and for this last one a third split event (node C, 95%HPD 2.04E5-3.54E6) led to CCK and HCoV-OC43/ BCoV I exclusive clusters. The RSCU distance of human and bovine CCK and SFTPA1 mRNAs were 0.136 and 0.221, respectively.

## Discussion

Codon usage optimization and deoptimization based on ΔCAI values for ORF1 nsps, observed for both the human enteric and respiratory tracts, might be a consequence of a balance between synthesis efficiency and fine-tuning codon usage adaptation to the new host codon usage after a bovine-to-human coronavirus spillover. Though these proteins are coded in the same ORF, the distinct roles they play during RNA replication and sgmRNAs transcription might demand not only different synthesis efficiencies but also, in some cases, compensatory or concerted codon usage evolution, as in the case of the proteases PLpro and 3C-like in nsps 3 and 5, respectively, which can process ORF1 polyprotein and release from it all subunits (Ziebuhr and Snijder, 2007).

The analysis of coronaviruses non-structural proteins of the replicase class allows deep phylogenies to be estimated (Snijder *et al.*, 2003) and results, thus, in a more representative range of evolutionary data to assess ancient virus/hosts relationship when associated to structural proteins data as herein.

Regarding the structural proteins, the different degrees of optimization and deoptimization found based on human enteric and respiratory tracts, besides the translation efficiency, might also be due to both an immune escape efficiency, as in the case of HE and S, as a lower CAI might lead to lower protein synthesis and consequently lower exposure to the immune system (Bahir *et al.*, 2009) and a fine-tuning codon adaptation leading to a more efficient receptor binding to the human tissues due to the primary S and accessory HE roles on this function (Popova and Zhang, 2002).

As seen in the MCC tree (Figure 3), the first split (Node A) of hosts (*H. sapiens sapiens/B. taurus taurus*) and HCoV-OC43/BCoV showed a 95% HPD from 1.54E8 to 2.44E5 years ago, ranging from the Kimmeridgian age of the Late Jurassic to the Middle Pleistocene.

Taking node A as a first split and thus as a consequence of a first contact between the codon usage of an ancestor coronavirus with the codon usage of an ancestor host, the lower limit (1.54E8 ya) brings the ancestor coronavirus codon usage status to an age compatible with the proposed ancient origin of coronaviruses as being 2.93E8 y.a. (Wertheim *et al.*, 2013), while the upper limit (2.44E5) is related to a time compatible with early humans, in agreement with the suggested interspecies transmission of a betacoronavirus prior to the HCoV-OC43/BCoV split (Vijgen *et al.*, 2006).

Such a large time span might be due to the lack of data from hosts and coronaviruses in between these upper and lower limits, but it places an ancestor betacoronavirus as coevolving with a diversity of dinosaurs (Langer *et al.*, 2010) in the Late Jurassic and reaching early humans with until unknown intermediate hosts during this large time span. It is worthy of note that this time span overlaps with the one found for node B (95% HPD 2.07E5-1.55E8), meaning that the first ancestor host/ ancestor betacoronavirus contact might have been stable for circa 150 million years before reaching early humans.

As for node C, the 95% HPD 2.04E5-3.54E6 embraces human evolution from *Australopithecus* spp to *H. sapiens sapiens* (McHenry, 1994), what could finally represent the first sign of BCoV spillover from an ancestor ruminant host to the human lineage after a first contact with the respiratory tract (represented by SFTPA1 in Figure 3).

The discrepancy of HPDs values, when compared to previous dates on the HCoV-OC43/BCoV split and the emergence of all coronaviruses, might be a consequence of both the use of full genomes data and the selection unit used in this survey, *i.e.*, codon usage, instead of subgenomic data based on nucleotide evolution as proposed by others (Vijgen *et al.*, 2005b; Vijaykrishna *et al.*, 2007; Munir and Cortey, 2015).

All coding regions/mRNAs from an ancestor coronavirus (except for HCoV-OC43 nsp7 in both respiratory and enteric human tracts, and for nsp15 on the human respiratory tract, ΔCAIs=0) experienced optimization or deoptimization, as suggested in Figure 1, probably after Node A (Figure 3). This process of codon usage evolution resulted in CAIs approaching the CAI limits for human coronaviruses as calculated herein (0.22-0.611 for the respiratory and 0.244-0.472 for the enteric tract) during codon usage evolution by natural selection, as shown in the *Nc* x %GC3s analysis (Figure 2). The association of data on fluctuations in codon usage optimization with analysis of the selection regime and a temporal analysis, both based on codon usage, as used in this investigation, might be of value for a deeper understanding of tempo and modes of viruses and hosts coevolution.

Having crossed the longer codon usage distance from the bovine to human respiratory tract (0.221) when compared to the enteric tract (0.136), HCoV-OC43 became a highly respiratory-specialized virus with high fitness to this new replication site and predating the proposed event around the year 1890 (Vijgen *et al.*, 2005b; Bidokhti *et al.*, 2013).

Though nsp14 is a coronavirus 3’-5’ exonuclease (Denison *et al.*, 2011), whose proofreading activity lowers the mutation rate of these viruses when compared to other RNA viruses, the mutant spectrum phenomenon is well documented in HCoV-OC43 and BCoV (Vabret *et al.*, 2006; Borucki *et al.*, 2013), and as a result, a plethora of synonymous mutations that power codon usage diversity is available for the optimization or deoptimization of codon usage in different genes via natural selection or drift as well.

An in important limitation to these arguments is that codon usage studies only allow speculations after virus attachment and entry, two processes intimately related to membrane receptor specificities that cannot be assessed in organisms for which at least gene data are not available. Also, the full set of interspecies jumps for the HCoV-OC43 ancestors has not been assessed here, as the focus was the proposed recent bovine-to-human spillover (Vijgen *et al.*, 2005b), and this might have limited the detection of further nodes of codon usage status split with coronaviruses and different hosts.

As a conclusion, via codon usage through natural selection resulting in immune escape balanced with protein synthesis efficiency, an ancestor coronavirus might have followed human evolution with no codon usage barrier fitness deep in the human lineage.
